# Overexpression of CCDC69 activates p14^ARF^/MDM2/p53 pathway and confers cisplatin sensitivity

**DOI:** 10.1186/s13048-019-0479-3

**Published:** 2019-01-16

**Authors:** Long Cui, Fang Zhou, Cui Chen, Chi Chiu Wang

**Affiliations:** 1 Department of Obstetrics and Gynaecology, Guangzhou Women and Children Hospital, Guangzhou, 511400 Guangdong China; 2 School of Nursing, The First Affiliated Hospital, Xuzhou Medical University, Xuzhou, China; 3 Intensive Care Unit, The First Affiliated Hospital, Xuzhou Medical University, Xuzhou, China; 4 Department of Obstetrics and Gynecology, Prince of Wales Hospital, The Chinese University of Hong Kong, Hong Kong, SAR China; 5 Reproduction and Development Laboratory, Li Ka Shing Institute of Health Sciences, The Chinese University of Hong Kong, Hong Kong, China; 6 School of Biomedical Sciences, The Chinese University of Hong Kong, Hong Kong, Shatin China

**Keywords:** Cell cycle, Chemoresistance,ovarian cancer, Apoptosis, p14ARF/MDM2/p53

## Abstract

**Objectives:**

The aim of the study is to explore the relationship between CCDC69 expression and resistance of ovarian cancer cells to cisplatin and reveal the underlying mechanism.

**Methods:**

One hundred thirty five ovarian cancer patients with intact chemo-response information from The Cancer Genome Atlas (TCGA) database were included and analyzed. Stable CCDC69 overexpressing 293 and ovarian cancer A2780 cell lines were established and subjected to examine cell apoptosis and cell cycle distribution using CCK-8 assay and flow cytometry. Cell cycle and apoptosis pathway were evaluated by immunoblots. Stability of p14^ARF^/MDM2/p53 pathway related proteins were determined by half-life analysis and ubiquitination experiments.

**Results:**

We found that *CCDC69* expression was significantly higher in chemo-sensitive groups compared with chemo-resistant groups from TCGA database. High *CCDC69* expression was associated longer survival. CCDC69 overexpressing 293 and A2780 cells with wildtype p53 and contributes to cisplatin sensitivity following treatment with cisplatin. We further found over-expression of CCDC69 activated p14^ARF^/MDM2/p53 pathway. Importantly, we also demonstrated that CCDC69 expression extended p53 and p14^ARF^ protein half-life and shortened MDM2 protein half-life. Ubiquitination assay revealing a decrease in p14 ubiquitination in CCDC69 over-expression cells comparing to cells expressing empty vector.

**Conclusions:**

It is tempting to conclude that targeting CCDC69 may play a role in cisplatin resistance.

**Electronic supplementary material:**

The online version of this article (10.1186/s13048-019-0479-3) contains supplementary material, which is available to authorized users.

## Introduction

Epithelial ovarian cancer (EOC) is the second most common and accounts for greatest number of death from gynecologic malignancies in the world wide [1, 2]. High-grade serous ovarian cancer (HGSC) is an aggressive subtypes and often associated with relatively unfavorable clinical outcomes [3]. Majority of advanced ovarian cancer patients initially responsive to chemotherapy; however, became increasingly ineffective and eventually results in treatment failure [4]. Thus, exploring and understanding the mechanisms responsible for chemo-resistance in ovarian cancer may improve the therapeutic outcomes.

Cisplatin has been the effective and widely used drugs against various human cancers, including ovarian cancer [5]. In response to cisplatin, p53, a key regulator of cell cycle checkpoints, activates a number of genes regulating cells cycle and/or apoptosis [6]. As transcriptional target of p53, p21 plays an important role in the maintenance of and G1 and G2 checkpoints following DNA damage such as cisplatin exposure [6–8]. In addition, p21 can regulate cell cycle progression dependent or independent of p53 following treatment with cisplatin [9, 10]. Previous reports have shown that abrogation of G2/M cell cycle checkpoint is related to enhancement of cisplatin-induced cytotoxicity [11, 12]. Expression of the p53 protein is posttranscriptionally regulated by MDM2. The MDM2 binds to p53 and blocking its activation domain [13], thus promoting degradation of p53 via ubiquitin-proteasome pathway [14, 15]. Under the DNA damage pressure, the p53 phosphorylation attenuates MDM2 binding ability and then leads to p53 accumulation [16]. Until recent years, investigators have identified another important regulator in this p53/MDM2 pathway, the cyclin-dependent kinase inhibitor p14^ARF^ (termed p19^ARF^ in the mouse) [17]. The p14^ARF^ protein is an alternative reading frame protein product of the CDKN2A/INK4A locus [18]. The other one is p16^INK4A^ protein [17]. It has been shown that the p14^ARF^ binds to the p53/MDM2 complex and inhibits MDM2-mediated degradation of p53 [19]. p14^ARF^ can inhibit cell cycle progression in both G1 and G2/M phases and/or cell apoptosis in a p53-dependent and independent manner [20, 21]. Furthermore, apoptosis stimulated by p14^ARF^ apoptosis is enhanced by loss of p21 mediated cell cycle checkpoint control [22].

Coiled-coil domain-containing (CCDC) proteins have variety of functions in regulating cell cycle progression and mediating apoptosis following DNA damage in eukaryotic cells [23, 24]. In recent years, several CCDC proteins have been widely studied as tumor suppressors in several types of cancer, such as breast and prostate cancers non-small cell lung cancer [25–27]. However, the studies on the role of coiled-coil domain-containing 69 (CCDC69) in ovarian cancer were limited. As one of these family members, *CCDC69* locates on 5q33.1 and is responsible for mitotic spindle and DNA replication [28]. In the present study, we found that *CCDC69* expression is significantly higher in chemo-sensitive groups compared with chemo-resistant groups from The Cancer Genome Atlas (TCGA) database. We also found women with high *CCDC69* expression was related to longer survival. To further elucidate the role of CCDC69, we then stably expressed CCDC69 in 293 cells and human ovarian cancer cell lines A2780 with functional p53. Our data showed that expression of CCDC69 abrogates G2/M arrest followed by apoptosis in these p53 wildtype cells. Importantly, we also demonstrated that CCDC69 expression extended p53 and p14^ARF^ protein half-life and shortened MDM2 protein half-life due to deubiquitination of p14^ARF^.

## Materials and methods

### Chemo-response and survival analysis using public datasets

TCGA clinical and *CCDC69* expression mRNA data were retrieved from published The Cancer Genome Atlas (TCGA) through the Computational Biology Center Portal (cBio): http://www.cbioportal.org/.The cgdsr extension package was used to execute the retrieval.

### Cell lines

Human ovarian cancer cell line A2780 was purchased from Sigma-Aldrich and routinely maintained in RPMI 1640 (Invitrogen) supplemented with heat-inactivated 10% (*v*/v) fetal bovine serum (Invitrigen) and 1% antibiotic-antimycotic. The human embryonic kidney cells 293 was purchased from ATCC (Bethesda, MD, USA). 293 cells were cultured in high-glucose DMEM (Invitrogen) containing 10% fetal bovine serum (FBS) (Invitrogen) and 100 U/ml of penicillin G/streptomycin. These mammalian cells were cultured in an incubator with an atmosphere of 37 °C in 5% CO^2^. Cell culture medium was refreshed every two days.

### Mutation analysis of *p53*

Polymerase chain reaction (PCR) amplification and direct sequencing were used to screen for DNA variations in the coding and the 5′ and 3′ flanking regions of p53. Primers for exons 5–8 are modified from Martin et al. [29]. Primers used for sequencing not shown Additional file 1: Table S1. All purified PCR products were sequenced for variations using an ABI PRISM® Big Dye™ Terminator version 3.1 Cycle sequencing kit (Applied Biosystems, Foster City, CA, USA) and an ABI 3100 automated sequencer (Applied Biosystems).

### Viral packaging and lentiviral transduction

For the production of lentiviral particles, 293 (1 × 10^6^) were transfected with CCDC69 lentiviral construct (pLenti-CCDC69-GFP) or control plasmid (pLenti-C-GFP, Origene, Bothell, WA, USA) together with pRSV-Rev and psPAX2 packaging vectors using Lipofectamine 2000 transfection reagent (Invitrogen). To generation stable cell lines, viral supernatant were harvested and filter through a 0.45 μm filter to remove cellular debris. The viral supernatant and 4.5 mL of fresh growth medium were added to plates and incubate the cells at 37 °C with 5% CO^2^ for 4 h. And then removed the transduction medium and added 10 mL of fresh growth medium. Incubated the cells for three more days in the presence of puromycin, and resistant colonies were selected for subsequent experiments.

### Cell viability assay

Cell viability was measured by MTT assay using the Cell Counting Kit-8 (CCK-8, Sigma-Aldrich) following the manufacturer’s instructions. The absorbance at 450 nm was measured in the microplate reader. The percentage of cell survival at each dose of cisplatin = Mean of A450 (drug-treated cells)/Mean of A450 (untreated cells). IC50 values were calculated using GraphPad Prism Software Version 5 (GraphPad Software Inc., CA, USA) and plotted in dose response curves.

### Flow cytometry analysis of cell apoptosis using Annexin V-FITC/PI staining

The experiments was performed according to the manual of Alexa Fluor® 488 Annexin V/Dead Cell Apoptosis Kit (Invitrogen) by flow cytometry. About 1 × 10^6^ cells were collected, washed with ice-cold PBS, and resuspended in binding buffer containing suitable amount of Annexin V-FITC. After 15 min of incubation in the dark at room temperature, the buffer was removed by centrifugation. The cells were then resuspended in reaction buffer containing propidium iodide (PI). Apoptosis was immediately detected by flow cytometry.

### Cell cycle assay

After the indicated treatments, cells were washed with cold PBS and harvested by centrifugation. Then, cells were re-suspended in 70% (*v*/v) cold ethanol and stored at − 20 °C overnight. After 30-min incubation with propidium iodide (PI) solution in the dark, cell cycle distribution was analyzed by Cytomics FC 500 (Beckman Coulter) flow cytometer. Results were calculated and visualized by Flow Jo software (version 10.0).

### Antibodies

Antibodies against CCDC69, p21 and p14^ARF^ were from Abcam (ab106692, ab109199 and ab3642). p53 Antibody (DO-1) and MDM2 antibody was purchased from Santa Cruz Biotechnology (Santa Cruz, CA, USA). HRP-conjugated anti-rabbit and anti-chicken IgG antibody were used as secondary antibody from Abcam (ab191866 and ab6877). GAPDH was used as an internal control for normalization of protein quantity.

### Real-time PCR

RNA was extracted with the RNeasy Mini Kit (Qiagen). cDNA was generated with PrimeScript cDNA Synthesis Kit (Takara). P21 mRNA expression levels were evaluated by real-time PCR using the ABI PRISM 7500 sequence detection system (Applied Biosystems, Foster City, CA). TaqMan™ Gene expression assays [cyclin dependent kinase inhibitor 1A (CDKN1A)/p21 (Hs00355782_m1)] were from Applied Biosystems. Quantification was based on a comparative ΔCt method using glyceraldehyde-3-phosphate dehydrogenase (GAPDH; Hs03929097_g1) as endogenous control. Each real-time RT-PCR was done in triplicate according to the manufacturer’s instructions.

### Half-life analysis

CCDC69 overexpressing 293 or A2780 cells and controls were incubated with cycloheximide (100 μg/mL; Sigma) to inhibit further protein synthesis. Following incubation for 30, 60, or 120 min, cells were harvested. Western blot was done as described above. The relative p53, MDM2, and p14^ARF^ levels were quantified by densitometry analysis using the ImageJ 1.410 image processing software.

### Immunoprecipitation

For immunoprecipitation/immunoblotting to detect ubiquitinated forms of p14^ARF^, cells treated with or without cisplatin in the presence of MG132 (20 μM). After incubations for 24 h, cells were lysed in RIPA buffer and protease and phosphatase inhibitor cocktail (Thermo Scientific). Protein lysates were precleared with protein G agarose beads (Millipore) for 1 h and then incubated with G-protein beads bound to p14^ARF^ rabbit polyclonal antibody (Abcam) for 2 h at 4 °C. Beads were washed 3 times in RIPA buffer. Protein was eluted from beads with 2 x SDS-β-mercaptoethanol sample buffer, boiled for 8 min and then loaded on polyacrylamide gels for SDS–PAGE as described above. Blots were blocked in BSA 5% (for phosphor antibodies) or non-fat dry milk 5% in TBS–Tween 0.1% for 1 h and then incubated with primary antibody. Samples were subjected to SDS-PAGE, followed by immunoblotting with indicated antibodies.

### Data statistical analysis

Data from at least three independent experiments were expressed as mean ± standard deviation (SD). The differences between two groups were analyzed using the *Student’s t test*. All analysis was performed using GraphPad Prism Software Version 5 (GraphPad Software Inc., La Jolla, CA, USA) at a two-sided 5% significance level.

## Results

### CCDC69 differentially expressed in chemo-sensitive and chemo-resistant groups

**Fig. 1:**
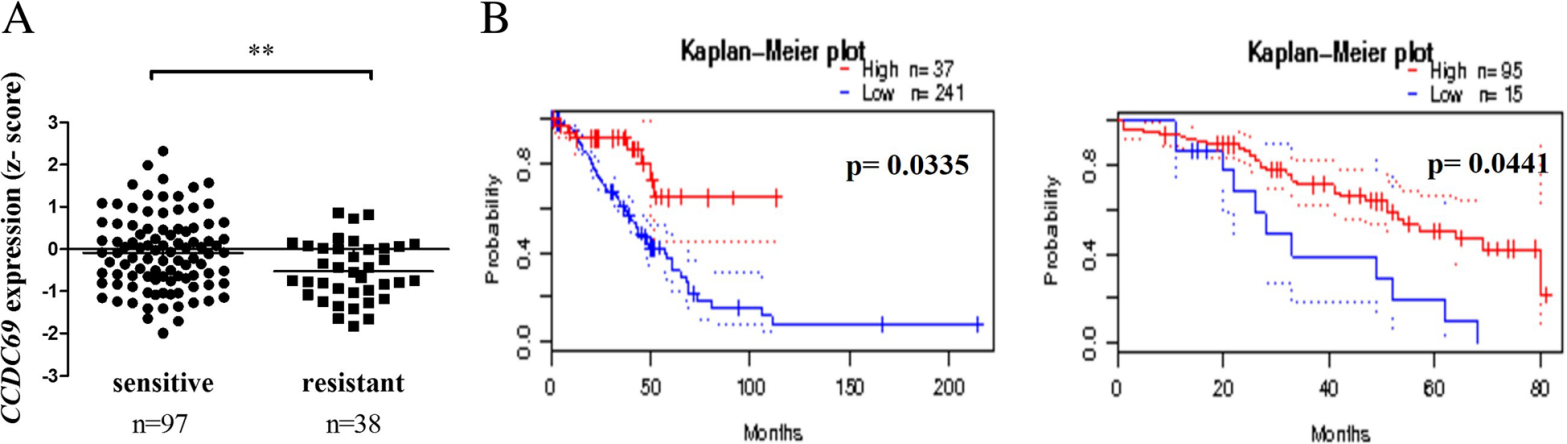
Higher *CCDC69* gene expression correlates with increased survival of ovarian cancer patients. **a**, dot plot for expression of *CCDC69* in chemo-sensitive groups and chemo-resistant group using TCGA database. **p < 0.01 versus resistant group (Student’s t-test). **b**, survival analysis for high-grade serous ovarian carcinoma patient using Gene Expression Omnibus (GEO) database

A total of 135 patients diagnosed with high-grade serous ovarian carcinoma with intact chemo-response information from The Cancer Genome Atlas (TCGA) provisional dataset were recruited into the study. The analysis revealed that *CCDC69* expression is significantly higher in chemo-sensitive groups compared with chemo-resistant groups (*p* = 0.0080) (Fig. 1a). The relationship between patient demographic variables and ovarian cancer chemo-response was analyzed (Table 1). Moreover, survival analysis using ovarian cancer microarray datasets (GSE9891 and GSE17260) indicated a significant association between high CCDC69 expression and better patient survival in the PrognoScan database [30–32] (Fig. 1 b and c**,** *p* = 0.033 and *p* = 0.044, respectively). These data indicate that CCDC69 expression confers to chemo-sensitive and plays a protective role in cancer cell survival.

**Table 1:** Characteristics of TCGA patients diagnosed with high-grade serous ovarian carcinoma

	Chemo-sensitive	Chemo-resistant	*p*-value
No. of patients	97	38	
FIGO Stage			*P* = 0.2011
II	9 (9.3)	1 (2.6)	
III	77 (79.4)	35 (92.1)	
IV	11 (11.3)	2 (5.3)	
WHO Grade			*P* = 0.6123
2	15 (15.5)	4 (10.5)	
3	81 (83.5)	33(86.8)	
Unknown	1 (1.0)	1(2.6)	
Residual disease			*P* = 0.0708
No Macroscopic disease	27 (27.8)	5 (13.2)	
1–10 mm	39 (40.2)	23 (60.5)	
11–20 mm	7 (7.2)	2 (5.3)	
> 20 mm	16 (16.5)	6 (15.8)	
NA	8 (8.2)	2 (5.3)	
Progression Free Survival(median, mons)	19.91 (3.3–87)	9.185 (2.3–15.2)	*P* < 0.0001
Overall Survival (median, mons)	41.03 (8.8–125.6)	32.835 (6.7–66)	*P* = 0.0047

### Overexpression of CCDC69 sensitizes 293 and A2780 cells to cisplatin

**Fig. 2:**
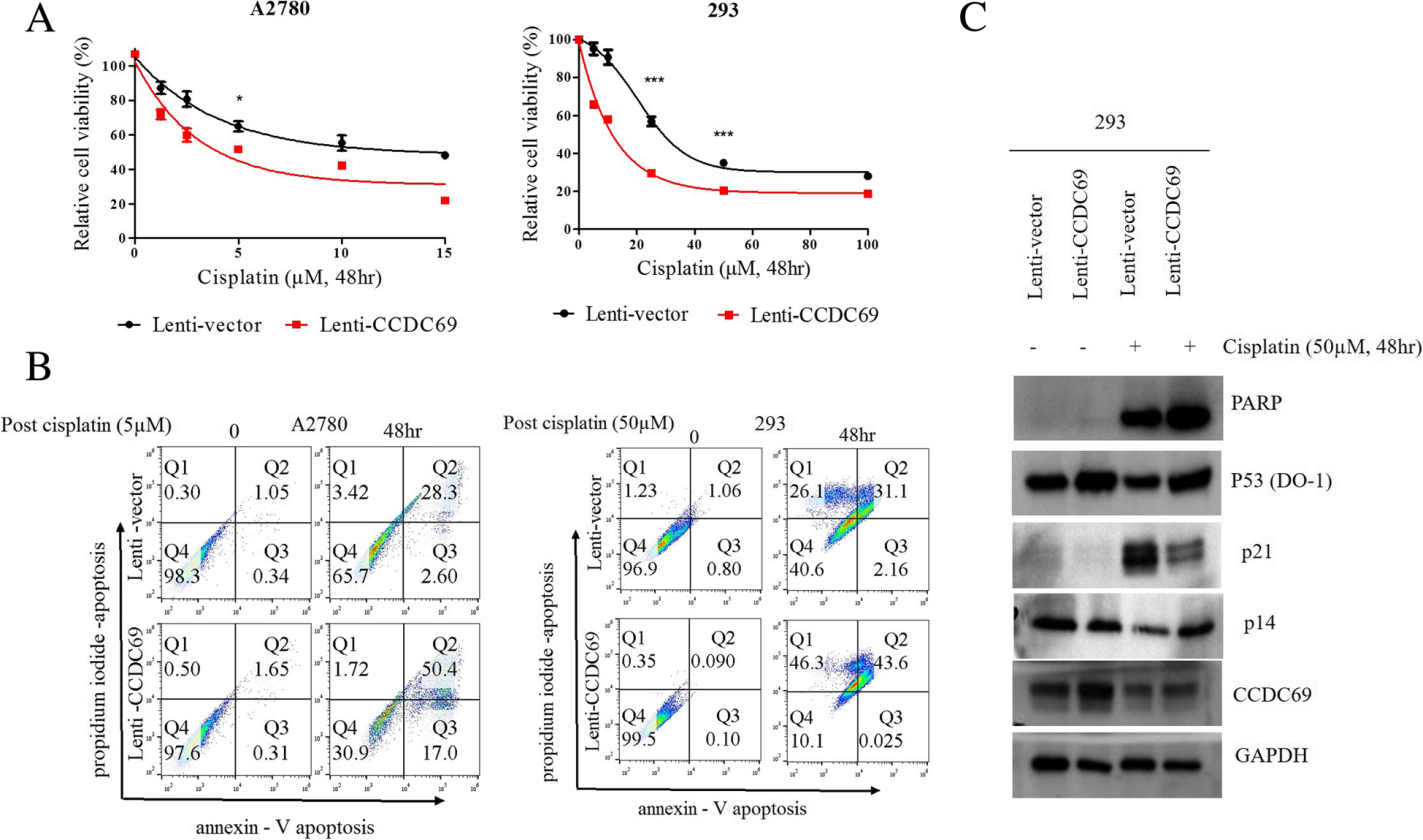
CCDC69 confers chemo-sensitivity in 293 and A2780 cells. **a.** Sensitization of cells to cisplatin after CCDC69 overexpression as revealed by the CCK-8 cytotoxicity assay. **b.** Apoptosis was analyzed by flow cytometry after annexin V and propidium iodide staining. Total apoptosis is the sum of the percentage of annexin V only and annexin V/propidium iodide stained cells. **c.** immunoblot analysis of CCDC69 and cleaved PARP in 293 cells after stable CCDC69 overexpression and treatments with cisplatin for 48 h. Loading control, GAPDH

To understand the role of *CCDC69* for cisplatin sensitivity to cells, A2780 and 293 cells were lentiviral transduced with a GFP tagged CCDC69 expression vector or with GFP as a negative control and cultured with puromycin (3 μg/ml) for 14 days. Exogenously expressed CCDC69 was detected by immunofluorescence staining (Data not shown). Immunoblot analysis confirmed that a higher CCDC69 expression in the CCDC69 overexpressing cells compared to those expressing an empty vector (Fig. 2c).

CCDC69 overexpressing 293 and A2780 cells showed an increase in cisplatin sensitivity compared to the cells expressing GFP (Fig. 2a**).** Moreover, an increased annexin V percentages of positive cells and higher levels of cleaved PARP were found in CCDC69 overexpressing 293 and A2780 cells compared to those expressing an empty vector in the presence of cisplatin treatment (Fig. 2b and c**).**

As a key molecule regulating apoptosis, we found that p53 protein levels were profoundly increased in CCDC69 overexpressing 293 cells compared to cells expressing empty vector treatment with or without cisplatin (Fig. 2c). Besides, DNA direct sequencing data showed no p53 mutations in A2780 and 293 cells. Collectively, these data indicate that CCDC69 plays an important role in enhancing cells to cisplatin-induced cell death.

### Downregulation of p21 in CCDC69 overexpressing 293 cells during cisplatin treatment arrest G2 arrest

**Fig. 3:**
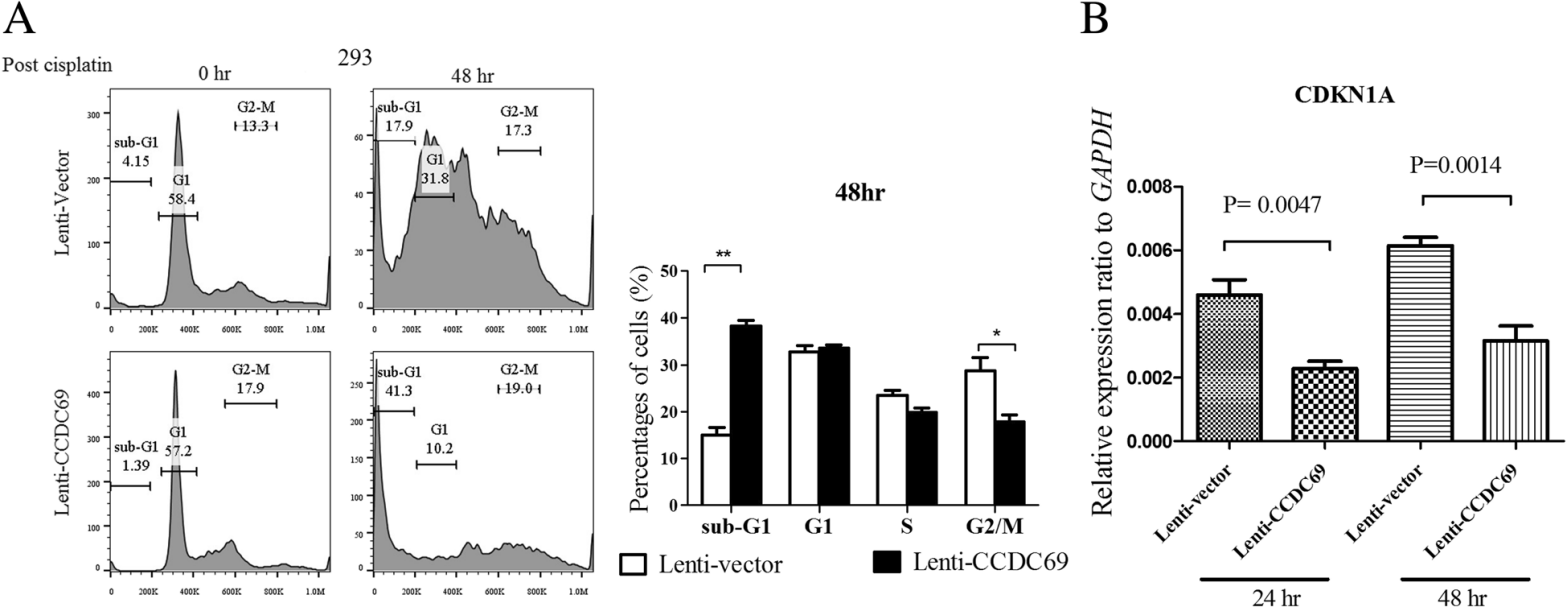
CCDC69 overexpressing cells showed abrogated G2/M arrest after cisplatin treatment. 293 wildtype and 293 CCDC69 overexpressing cells were treated with 50 μM cisplatin for 48 h, and then cell cycle was analyzed by flow cytometry. Data represent the mean and the standard deviation from three independent experiments. *p < 0.05 and **p < 0.01 versus cisplatin-treated 293 wildtype cells (Student’s t-test)

As one of the downstream target of p53, we next assessed the expression of p21 by Western blot. The data showed that p21 was marked decreased in CCDC69 overexpressing 293 cells than cells expressing empty vector (Fig. 2c). We further determine the cell cycle phase distribution in CCDC69 overexpressing 293 cells and cells expressing empty vector using flow cytometry. We found that CCDC69 overexpressing 293 cells had significant lower percentages of G2/M phase (Fig. 3). Consistent with apoptotic experiments, we found obvious accumulation of CCDC69 overexpressing cells at sub-G1 (Fig. 3a), which is a clear indicator of apoptosis.

It has been reported that stabilized or accumulated p53 without induction of p21 protein level, suggesting that the induced p53 was transcriptionally inactive [33–35]. We therefore investigated the expression of p21 mRNA in both CCDC69 overexpressing 293 cells and cells expressing empty vector. An increase in p21 mRNA expression was seen at 8 h and 24 h following treatment with cisplatin (Fig. 3b). Collectively, these results indicate that abrogated cell cycle phase may due to p53-independent downregulation of p21 following treatment with cisplatin.

### CCDC69 regulates p53/MDM2/p14^ARF^ signaling pathway

To explain the accumulation of p53, the p14^ARF^/MDM2/p53 pathway related proteins were evaluated. We found marked reduction in the expression of p14^ARF^ in CCDC69 overexpressing 293 and A2780 cells. These results indicate that CCDC69 enhances the accumulation of p53 may result from inhibition of p14^ARF^ after overexpression of CCDC69.

**Fig. 4:**
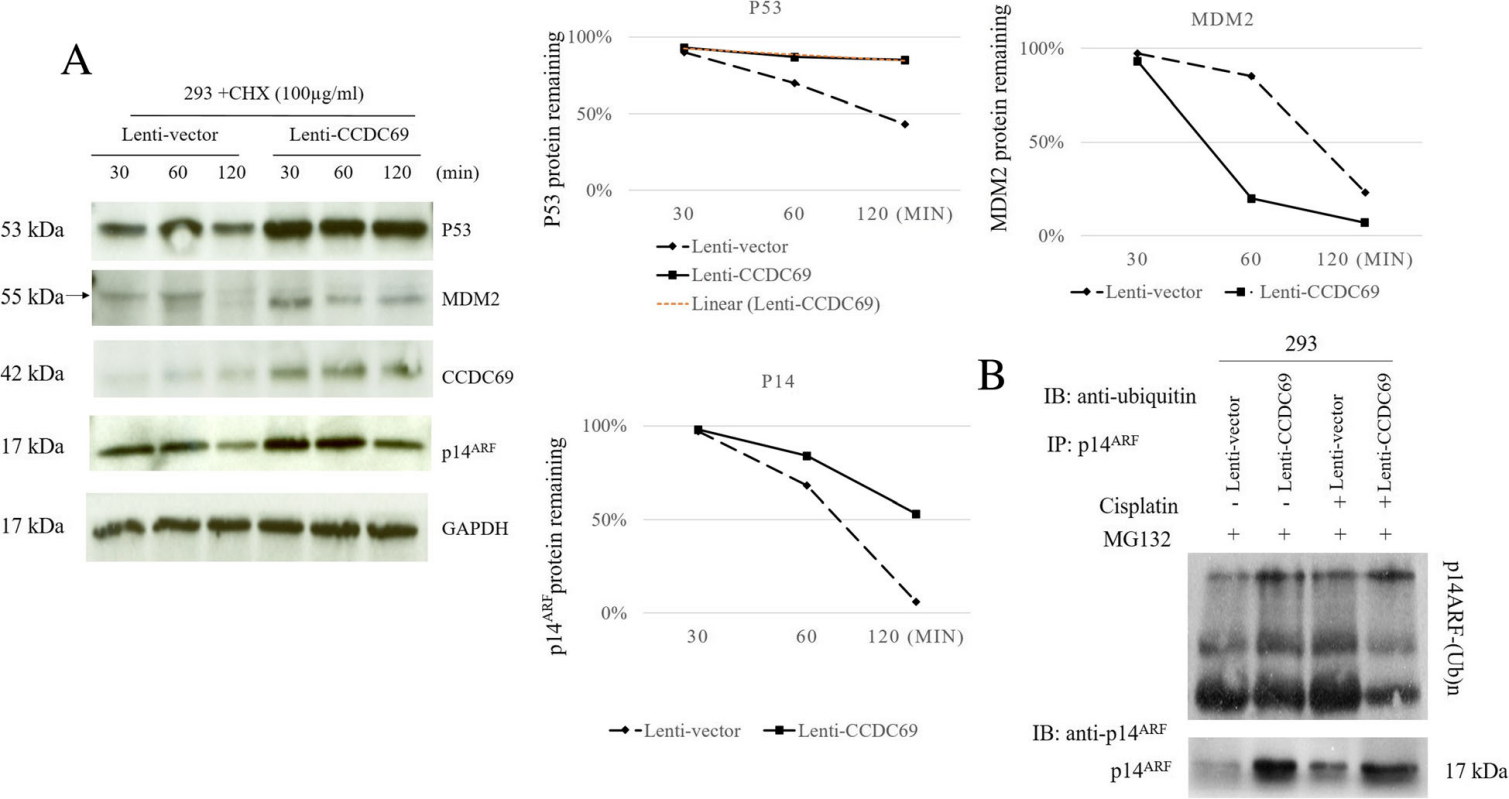
CCDC69 regulates p53, MDM2, and p14^ARF^ stability. **a**. 293 wildtype and 293 CCDC69 overexpressing cells were treated with 100 μg/mL cycloheximide (CHX) for the indicated periods, followed by Western blot using antibody against p53, MDM2, p14^ARF^, or CCDC69, with GAPDH as the loading control. The relative p53, MDM2, and p14^ARF^ levels were quantified by densitometry analysis (right). **b**. Ubiquitylation assay revealing a gradual increase in p14^ARF^ ubiquitylation from CCDC69 overexpression 293 cells. IP, immunoprecipitation; IB, immunoblot

Therefore, to further determine whether p14 promoted MDM2 degradation is involved in the accumulation of p53 in the presence of CCDC69, we assessed the expression of p14, p53 and MDM2 after treatment with the inhibitor of protein biosynthesis CHX (cycloheximide) (100 μg/ml) for various lengths of time (Fig. 4a). As it was shown in Fig. 4a, the data showed that CCDC69 overexpression significantly extended the half-life of p53 and p14^ARF^, on the contrary, shortened the half-life of MDM2 (Fig. 4a), suggesting that CCDC69 promotes the accumulation of p53 through activating p14^ARF^ while inactivating MDM2 signaling to sustain p53 and p14^ARF^ expression during cisplatin exposure. Furthermore, ubiquitination assay revealing a decrease in p14 ubiquitination in CCDC69 overexpression cells comparing to cells expressing empty vector (Fig. 4b).

## Discussion

The p14^ARF^/MDM2/p53 pathway has been well established signaling axis in determining cancer cells apoptosis/quiescence or senescence [36]. It is worth noting that p14 could also induce p53-independent apoptosis [20, 21]. Furthermore, p14 triggered apoptosis is enhanced by loss of p21 mediated cell cycle checkpoint by p53-mediated mitochondrial apoptotic cascade [22, 37]. In our study, immunoblots analyses in p53 wildtype cells indirectly suggested that p14 is dispensable for accelerating cell death after induction of CCDC69 following treatment with cisplatin. Most intriguingly, it was reported recently that p53 aggregation could sensitize cancer cells to platinum treatment [38]. The most likely explanation is that these controversial findings may come from different populations of cancer cells properties. Therefore, the exact nature of the molecular mechanisms involved in these processes need for further investigation in the future.

In our study, we demonstrated that CCDC69 expressing cells abrogated G2 arrest in the reduction expression of p21. Considering that p14 could induce cell cycle arrest in both G1 and G2 phases in a p53-dependent and independent manner [39–41]. One of the explanation is that p21 might be the major cause for G2 cell cycle arrest in response to CCDC69 induction following treatments with cisplatin. Moreover, we showed that reduction of p21 expression in an entirely p53-independent fashion, as the p21 mRNA was found to be transcriptionally inactive. We excluded genetic mutation for p53 inactivation and learnt that many tumors without p53 mutation still harbor a transcriptionally inactive form of p53 [42, 43]. These findings raise the question of the importance of cell cycle variations in cancer cells.

Numerous studies have demonstrated that the tumor suppressor protein p14^ARF^ can stabilize and activate p53 by inhibiting the E3 ligase activity and nuclear export of MDM2, which is followed by formation of the p14ARF-MDM2-p53 ternary complex [44–46]. Based on the extended half-life and decreased ubiquitination level of p14^ARF^ in the CCDC69 overexpressing cells, we concluded that CCDC69 was able to activate p14^ARF^ by increasing stability of the p14^ARF^ protein, which, in turn, promotes activation p53 pathway. Based on above findings, CCDC69 mediates stability of p14^ARF^ and regulates cell cycle and apoptosis response to chemotherapy. Therefore, CCDC69 may be a promising target for therapeutic application in cancer. Although p53 mutations are most frequently occurred in cancer [47, 48], researchers had defined an activity of p14^ARF^ in sensitizing p53 mutated osteosarcoma cells to cisplatin [49]. However, we did not assess the ubiquitination status of p53 and MDM2. Moreover, the physical interactions between p14 and CCDC69 should be further investigated in the future.

In summary, we present data showing that the expression of CCDC69 in chemosensitive groups was significant higher than chemoresistance groups. In addition, the high expression of *CCDC69* was associated with better survival based on publicly available databases. Furthermore, CCDC69 could activate the p14^ARF^/MDM2/p53 signaling pathway, resulting in cancer cell apoptosis. Thus, our study provide knowledge about the role of CCDC69 in chemoresistance of ovarian cancer and provide us further clue develop new therapeutic strategy for ovarian cancer patients.

## Conclusion

In conclude, CCDC69 could be play important roles in regulating chemoresistance through activation of p14^ARF^/MDM2/p53 signaling pathway.

## Additional file

Additional file 1: **Table S1.** Primers and PCR conditions used for amplification of *p53 (DOCX 16 kb)*

## Abbreviations

BSA: Bovine serum albumin
cBio: computational biology center portal
CCDC: Coiled coil domain containing
CCDC69: Coiled coil domain containing 69
CCK-8: Cell Counting Kit-8
CHX: Cycloheximide
DMEM: Dulbecco modified eagle medium
EOC: Epithelial ovarian cancer
FBS: Fetal bovine serum
GAPDH: Glyceraldehyde-3-phosphate dehydrogenase
HGSC: High-grade serous ovarian cancer
IB: Immunoblotting
IP: Immunoprecipitation
MDM2: Mouse double minute 2 homolog
MTT: 3-(4,5-Dimethylthiazol-2-yl)-2,5-diphenyltetrazolium bromide
PCR: Polymerase chain reaction
PI: Propidium iodide
SDS-PAGE: Sodium dodecyl sulfate polyacrylamide gel electrophoresis
TCGA: The Cancer Genome Atlas
