# Supplementary material for: Overexpression of CCDC69 activates p14ARF/MDM2/p53 pathway and confers cisplatin sensitivity
Source: J Ovarian Res. 2019 Jan 16;12:4. doi: 10.1186/s13048-019-0479-3 (PMC6334460; doi:10.1186/s13048-019-0479-3)
Supplement: Supplementary file 1 — Table S1. Primers and PCR conditions used for amplification of p53 (DOCX 16 kb) [file 13048_2019_479_MOESM1_ESM.docx]

| **Additional file 1: Table S1: Primers and PCR conditions used for amplification of *p53*** | | | | | | |
| --- | --- | --- | --- | --- | --- | --- |
| **Primers ^a^** | **Sequence (5' to 3' )** | |  | **Product size** | | **Conditions** |
|  |  | |  |  | |  |
| *P53*-2&3F | | ggatccccacttttcctctt |  | | 208 | 1mM MgCl_2_,  5% DMSO;  Tm 65°C |
| *P53*-2&3R | | agcatcaaatcatccattgc |  | |  |  |
| *P53* -4F | | gacctggtcctctgactgct |  | | 380 | 1mM MgCl_2_,  5% DMSO;  Tm 60°C |
| *P53* -4R | | atacggccaggcattgaagt |  | |  |  |
| *P53* -5&6F | | tgccctgactttcaactctgt |  | 466 | | 1mM MgCl_2_,  Tm 65°C |
| *P53* -5&6R | | ttaacccctcctcccagaga |  |  |  |  |
| *P53* -7F | | CGACAGAGCGAGATTCCATC |  | 420 | | 1.5mM MgCl_2_,  5% DMSO;  Tm 55°C |
| *P53* -7R | | TGTGATGAGAGGTGGATGGG |  |  |  |  |
| *P53* -8&9F | | caagggtggttgggagtaga |  | 295 | | 1.5mM MgCl_2_,  5% DMSO;  Tm 62°C |
| *P53* -8&9R | | acttgataagaggtcccaag |  |  |  |  |
| *P53 -*10F | | atgttgcttttgatccgtca |  | 332 | | 1mM MgCl_2_,  Tm 66°C |
| *P53* -10R | | ctttccaacctaggaaggca |  |  |  |  |
| *P53* -11F | | GCATTGGTCAGGGAAAAGGG |  | 420 | | 1mM MgCl_2_,  Tm 55°C |
| *P53* -11R | | CCGGGACAAAGCAAATGGAA |  |  |  |  |
|  | |  |  |  | |  |
| ^a^: Primers were designed from reference sequence with GenBank accession no. NM_000546.5NM; F: forward; R: reverse | | | | | | |
